# Author index to Volume 82

**DOI:** 10.1054/bjoc.2000.1284

**Published:** 2000-05-18


					Abel A 1939
Aceto G 348
Adams S 1650
Adlercreutz H 1107
Agsteribbe E 1474
A'Hern R 568
Ahern R 812
Ahner R 1249
Ahren A-M 1332
Ahrens W 227
Ajayi L 1671
Ajioka Y 1689
Akalin I
.
249 (L)
Akechi T 800
Alarcon-Gonzalez P 1145
Alexander F 1353
Alexander FE 1117, 1571
Alhava E 900
Alhava EM 2017
Allal AS 1131
Allalunis-Turner MJ 635
Allan D 74
Allegra CJ 560
Allen DS 1577
Allen-Mersh TG 1004
Alvarez-Mon M 1611 (L)
Ambrosetti P 1131
Amnan K 157
Amo-Takyi BK 1407
Anchisi S 1131
Anderson H 1261
Anderson V 705
Anderson VA 255
Ando Y 497 (L)
Angerson WJ 74
Angius A 553
Angstrom T 1332
Aninat-Meyer M 65
Anthony DA 1932
Antoine M 412
Antonopoulos MJ 300
Anttila MA 1983
Aozasa K 1446
Araki K 1892
Ariga N 518
Armatis P 1724
Armstrong AA 1117
Armstrong B 1344
Arnett TR 1459
Aromaa A 1107
Arteta B 953
Artuc M 1453
Asada M 1945
Asakawa S 1801
Asakura H 1689
Asanoma K 459
Ascari E 1254
Ashcroft L 1158
Ashihara T 1327
Asmar L 529
Asosingh K 953
Astrom M 1387
Atagi S 418
Atkin SL 1312
Atra A 1396
Aubele M 1204
Auersperg N 1415
Austrian Hereditary Breast and
Ovarian Cancer Group 1249
Auterith A 1249
Autier P 1887
Azzarelli A 1271
Baak JPA 368
Babylon A 399
Badie C 642
Bae MH 385
Bae SK 385
Baguley BC 966
Bailey NP 93
Bak M 339
Balli F 702
Balzi M 178
Banno Y 16
Barachini S 905
Barbarano L 1254
Barnes DM 1145
Barozzi C 865
Barozzi P 702
Barrett JC 459
Barrett-Lee PJ 93
Barroso A 1266
Barshishat M 195
Bartolomei M 295
Bartsch G 39
Bartsch H 763
Baser ME 998 (L)
Bassano L 1732
Bataille V 247 (L)
Battista P 348
Bay BH 1198
Bayerdorffer E 1795
Beale PJ 436
Beard SM 81
Becciolini A 178
Becker M 1844
Beijnen JH 1539
Bell J 1111
Bell JA 1163
Bellefqih 871
Benard J 871
Benini E 270
Benitez J 1266
Benoy I 1896 (L)
Bergamaschi D 1732
Berger P 85
Bergh J 777
Bergman F 1332
Bergonzi C 1254
Bernaudin J-F 412
Bernstein ED 1611 (L)
Bertram B 629
Betts DR 1239
Bevilacqua G 905
Bianco AR 1772
Bianco T 131
Bibby MC 1925
Bicknell R 161, 749
Bieche I 142
Bieri S 1131
Biesova Z 1808
Bigioni M 480
Bignell G 151
Biomarkers Ad-hoc Group of the
United Kingdom Coordinating
Committee on Cancer
Research 1625, 1627
Birch JM 1571
Black DM 705
Black MH 361
Black MJ 757
Blair V 1571
Blamey RW 1163
Blanco I 535
Blijham GH 1914
Blomqvist CP 777
Boavida M 323
Bocci G 905
Bocciolone L 295
Boddy AV 1519
Bodin L 1387
Bodingbauer Y 1149
Boffetta P 227, 1344
Bogdanova TI 315
Bohles H 399
Bojunga J 1650
Bolton E 167
Bombled J 818
Bonaiti-Pellie C 1939
Bonilla F 1183
Borel Rinkes IHM 1017
Borriello A 1171
Bos AME 1920
Bosch A 151
Bosetti C 204
Boshoff C 702, 812
Bosq J 1614 (L)
Bounacer A 308
Bourhis J 642
Bowrey PF 167
Boxall F 436
Boyle P 1887
Bras-Goncalves RA 913
Braybrooke JP 1759, 1776
Breitkreutz R 399
Brennan P 1344
Bressac-de Paillerets B 818,
871, 1939
Bribes E 1965
Bridge J 823
Brinton LA 1600
Brock CS 608
Bromsztyk K 1041
Brown KW 323
Brown N 844
Brown R 392
Brugieres L 1939
Brugnatelli S 1254
Brunet J 535
Brussen J 1577
Buchs N 858
Buckley CH 760
Buckner J 1371
Budai B 492
Budroni M 553
Bullard J 1111
Bullbrook RD 1577
Bult P 381
Bunch KJ 1339
Bundred NJ 354
Bunnell H 315
Burkle A 629
Buskens E 1017
Butler LM 131
Butler SA 1553
Buttner B 1844
Buzdar AU 529
Cai J 1317
Caillou B 308
Calvert AH 1519
Cama A 348
Cameron DW 446
Campbell NC 1863
Camplejohn RS 1145
Capella G 535
Caput D 823
Carbone R 1171
Carella R 865
Carelle N 142
Carinci F 1613 (L)
Carls PF 1613 (L)
Carreras J 20
Carss A 315
Carter R 1103
Carter RL 1396
Cartwright RA 1117
Casellas P 1965
Cassidy J 1863
Castelao JE 1364
Casu G 553
Cebrian A 1266
Author index to Volume 82
Key to abbreviations:
(BR) Book reviews; (L) Letters to the editors
2024
British Journal of Cancer (2000) 82(12), 2024-2031
(c) 2000 Cancer Research Campaign
doi: 10.1054/ bjoc.2000.1284, available online at http://www.idealibrary.com on
Cecic I 1835
Cerundolo V 1058
Cha HJ 385
Chakraborty S 1682
Chandra S 1764
Chang J 538
Chang JT-C 1953
Chang S-H 1871
Chaplin DJ 1835
Charles AK 323
Chave HS 124
Chen C-Y 852
Chen HJ 1694
Chen K-W 852
Chen TH-H 1871
Chen Y 2002
Cheng A-J 1953
Cherstvoy ED 315
Chessa L 1945
Cheyns P 1123
Chi Y 657
Chiappetta G 315
Childers SR 1223
Chinje EC 651
Chiron M 642
Choi HS 1403
Chompret A 1939
Christodoulos K 1759
Cianchi F 178
Claeskens A 1123
Clarkson P 1348
Climent F 20
Cline JM 1223
Coates M 1344
Coates RJ 1600
Cocorocchio E 524
Cocquyt V 823
Coderre JA 1764
Cohen B 705
Cohen BB 1247
Cohen Tervaert JW 472
Cole S 1925
Coleman G 568
Coleman MP 1111
Coleman N 1218
Coleman RE 858, 1186, 1547
Colin D 1344
Collan Y 1656
Collette L 283
Collins HM 960
Collins VP 543, 1218
Colombo N 295, 616
Colston KW 1459
Conti E 204
Cook AM 1959
Cookson S 1009
Cools M 1123
Cooper RA 1177
Cooperative Group of Study and
Treatment of Multiple
Myeloma 1254
Cornelisse CJ 151
Corsini C 524
Cossu A 553
Costa-Pereira AP 1827
Cotter TG 1827
Cotterill SJ 1636
Couper R 945
Couturier D 871
Cowled PA 131
Cox G 1427
Cranston DW 161
Craven J 213
Crayford T 742
Cremonesi M 295
Crew J 161
Crognale S 348
Cronauer MV 39
Cucci F 702
Culig Z 39
Cummings J 1629
Cummings MC 1204
Cummins R 1740
Curia MC 348
Curmi PA 142
Curtin NJ 924
Curtis D 330
Cuschieri A 213
Cuzick J 1348
Czajka A 1485
Czarnotta B 1851
Da
browska A 1485
Daidone MG 270
Dalgleish AG 1009, 1619
Dalibor DV 1757 (L)
Damecour E 263
Damiano V 1772
Danel C 412
Danesi R 905
Danielsen T 1528
Dann JL 705
Dansonka-Mieszkowska A 579
Darnton SJ 1510
Das H 1682
David AS 742
Davidson RH 705
Davidson SE 1177
Davies J 760
Davies MM 1004
Davies SM 753
Davies T 208
de Benedetti A 161
de Boeck G 1000
de Bruijn EA 1000
De Cicco C 295
de Graaf H 1920
De Greef C 953
de Jong FH 112
de Jong JS 368
de Leede G 806
de Leij LFMH 472
De Lena D 560
De Lorenzo S 1772
De Mey R 705
De Mulder PHM 772
De Paepe A 823
De Palo G 270
De Paoli A 1254
de Reijke TM 283
de Schipper FA 1421
De Stavola BL 1577
De Veerman M 953
De Vos F 291
de Vries EGE 767, 1920
de Wilt JHW 973, 1000
de Winkel A 492
De Wolf-Peeters C 823
Dearnaley DP 1959
Declerck PJ 1702
Dedola MF 553
Dejosez M 1851
Del Governatore M 56
del Re EC 1317
Del Tacca M 905
Delfini C 1254
Delia D 1945
Della Ragione F 1171
Della Torre G 1271
Dellian M 1513
Delmas PD 858
Demidchik EP 315
Denis MG 1283
Denison U 1138
Denley H 1163
Depisch D 98
D'Errico A 865
Desai SB 1312
Desjardins L 818
Dessarps-Freichey F 1939
Dettke M 441
Devereux S 278
Devesa SS 1875
Devilee P 151
Dhaene K 1051
Di Biase AR 702
Di Liberti G 1732
Di Paolo A 905
Di Simone M 865
Di Stasi M 1254
Diamandis EP 361
Diel IJ 1381
Dietel M 65, 488
Dieumegard B 871
Dillner J 1332
D'Incalci M 524, 1732
Dinjens WNM 1510
Dirix LY 1896 (L)
Dixon JE 666
Dixon JM 1629
Dixon RM 1776
Dobbs N 1895 (L)
Dobrovic A 131
Dobson LS 1547
Dohjima T 16
Doihara H 46
Dominguez F 584
Dominguez G 1183
Dondon M-G 1939
Dono K 1211
Double JA 1925
Dougal M 1261
Droge W 399
Du-Villard JA 308
Duddy PM 1145
Dumont JE 315
Dunbar PR 1058
Duncan I 1348
Durany N 20
Dutch Immunotherapy Working
Party 772
Dutrillaux B 913
Duval N 263
Eatock MM 1932
Eaton N 1103
Ebert MPA 1795
Ebert W 782
Eden OB 1396, 1571
Edler L 399
Edwards DR 1612 (L)
Eeles R 499 (BR)
Eeles RA 1145
Egashira A 1892
Eggermont AMM 973, 1000
Eguchi H 1211
Eguchi K 104
Eisen T 812
Eiser JR 1605
Ekert H 255
Ekstrom C 1561
Ekstrom TJ 1561
El-Abassi M 1932
Elbe B 1844
Elias E 742
Elliott AM 1863
Elliott P 1103
Ellis IO 1163
Elstner E 452
Engers R 1063
Enomoto T 1446
EORTC Genitourinary Group
283
Eppinga P 806
Epstein J 1433
Erba E 1732
Erdkamp FL 1914
Eriksson T 1561
Erjavec Z 806
Erselcan T 777
Esiri MM 1441
Eskelinen M 900
Eskelinen MJ 2017
Espana P 1183
Esposito DL 348
Esslimani M 1965
Ethier SP 666
Etienne MC 171
Ettore F 171
Evans DGR 998 (L)
Evans H 608
Evans MF 424
Evans TRJ 1932
Ezaki T 1814
Fabbro D 1063
Fabbrocini A 1772
Fader DJ 657
Faircloth G 1732
Fallowfield L 1783
Fan Z 1993
Fang M 1993
Faragher EB 354
Faraoni P 178
Farmery SM 1233
Farris A 553
Fayers PM 213
Fazio F 616
Fears TR 1875
Fehringer M 794
Fei G 1795
Feifel G 794
Felberbauer FX 1758 (L)
Feleszko W 1485
Fentiman IS 1577
Fenton BM 937
Ferguson-Smith MA 1218
Fernandez PL 20
Feroze F 538
Ferrero JM 171
Author index 2025
British Journal of Cancer (2000) 82(12), 2024-2031
(c) 2000 Cancer Research Campaign
Ferrucci PF 524
Ferruzzi L 865
Feunteun J 1939
Ficari F 348
Fichtner I 1844
Fielding J 213
Fiorentino M 865
Fiorenza M 295
Fleischmann E 1249
Fogli S 905
Fokkema E 767
Fong KM 1191
Fontanella E 1945
Fordyce A 705
Formento JL 171
Fossa SD 737
Foulkes WD 757, 1646
Fox SB 844
Franceschi S 204, 1860
Francoual M 171
Franko AJ 635
Frati L 1945
Fraumeni Jr JF 718, 1875
Frayling IM 827
Frebourg T 1939
Freedman LS 213
Freeland J 1117
Frelinger JG 937
Freshney RI 881
Friedmann A 1249
Friery OP 1469
Frigerio L 616
Frisch RE 726
Frout D 263
Fuggle S 161
Fujii A 404
Fukuda J 1415
Furuse K 418
Fusco A 315
Fusenig NE 591
Gabbert HE 1063, 1851
Gaborieau V 227
Gago-Dominguez M 1364
Galanis E 1371
Galettis P 966
Gallagher CJ 2024
Gallagher R 1469
Galligan ES 1469
Gallinger S 1646
Gallus S 1860
Gamallo C 1183
Gambini C 1171
Gammon MD 1600
Ganesan TS 1759, 1776
Ganly I 392
Garbe C 1149
Garcia JM 1183
Gardes M 1939
Garnero P 858
Garnham J 330
Gatter K 1427
Gatter KC 844, 1441
Gebhardt MC 1327
Gee JMW 501
Geerts ML 823
Gellert K 65
George L 731
Geradts J 1191
Gerharz CD 1063, 1851
Gerrard M 1396
Gerth R 85
Gertsch P 1131
Ghadirian P 757, 1646
Ghielmini M 524
Ghilchik MW 492
Giannoudis A 424
Giatromanolaki A 844, 1427
Giermasz A 1485
Gilchrist R 1145
Giles GG 1887
Gillespie AM 1186, 1393
Giordani L 1171
Giordano M 1254
Godard V 412
Godber T 255
Goepel JR 1186
Gokhale DA 1117
Gol
/a
b J 1485
Goldstone AH 278
Gonthier M-P 142
Gonzalez I 535
Gonzalez R 1183
Gonzalez-Aguilera JJ 535
Gore ME 812
Gorin I 818
Gough AC 124
Gourgou S 1965
Grabau DA 339
Grabbe J 1453
Graf AH 1138
Graham DI 74
Grana C 295, 616
Grandjouan S 871
Gray IC 1671
Gray NE 1671
Gray SG 1561
Greenberg RS 718
Gregory RK 1907
Grenier J 1965
Griffin MJ 1519
Griffiths JR 2009
Grigioni WF 865
Groen HJM 767, 806
Groenendijk RPR 381
Groenewegen G 772
Grohs JG 85
Groot K 1724
Grybos M 621
Guerry M 1614 (L)
Guillou PJ 1233
Gullick WJ 1163
Gullu IH 249 (L)
Gunther K 1407
Gupta RK 2024
Gustafson TA 683
Gusterson B 568
Habib FK 186
Hack V 399
Haddada H 642
Hagmuller E 399
Hahn L-J 1871
Haider K 98
Hallmans G 1332
Halm U 1013
Halmos G 1724
Hambley TW 966
Hamblin MR 56
Hamburger AW 683
Hamel N 757, 1646
Hamidi S 1433
Hamilton JA 1671
Han C 1759
Hancock BW 1186, 1393, 1547
Handt S 1407
Hann IM 1396
Hansen S 339
Hara A 467
Harada M 104
Harding C 354
Harland C 1605
Harper P 1158
Harris AL 161, 651, 844, 1427,
1441, 1759, 1776, 1895 (L)
Hartmann O 251
Hartschuh W 591
Hasan T 56
Hasegawa K 1682
Hashiguchi S 1327
Hattori Y 657
Hausmaninger H 1138
Hayashi S 838
Hayashi Y 1801
Hayes RB 718
Hayward JL 1577
Hebert F 1724
Heinrich J 227
Heinzer H 246 (L)
Helbich T 1249
Helfrich W 472
Heliovaara M 1107
Helmbach H 488
Helzlsouer KJ 731
Henderson BE 1867
Hendrich S 1879
Hennig E 1041
Henriksson R 246 (L)
Henry-Amar M 263
Henz BM 1453
Heo SC 1403
Herfarth C 157
Heriot AG 1009
Hernberg M 777
Herrington CS 424
Hibi T 1717, 1814
Higashi T 833
Higashiyama M 374
Hill C 1614 (L)
Hillen HF 1914
Hipfel R 1149
Hirabayashi K 981
Hiraki S 104
Hiraki Y 104
Hirasawa Y 1327
Hirata M 1327
Hirst DG 1469
Ho L 1348
Ho S 497 (L)
Hobisch A 39
Hobson R 1396
Hoekman K 772
Hofler H 315, 1204
Hogel J 118
Holdaway IM 241
Hollstein M 763
Holm E 399
Holm S 1561
Holting T 157
Holzer G 85
Hombauer H 1290
Honma T 1689
Hoodfar E 705
Hooper ML 1247
Hoover RN 718, 1600
Hopewell JW 1764
Horii A 1801
Horiuchi S 891
Horsburgh T 186
Hortobagyi G 529
Horwich A 1959
Hosaka T 1677
Hoshi M 1801
Hosoe S 418
Houlston RS 568
Howard GCW 136
Howarth GS 945
Howe FA 2009
Howell A 354
Howell SB 34
Howell SJ 789
Hsieh T-J 1035
Hsu C-H 1035
Hsu C-P 1480
Hsueh S-F 1480
Hua D 1939
Huang C-I 374
Huang TH-M 514
Huckriede A 1474
Huddart RA 1959
Hudis C 1897
Hughes AN 1519
Hughes CM 1469
Hughes-Fulford M 2002
Huland E 246 (L)
Hunter RD 1177
Hutzler P 1204
Ichikawa T 838
Ichimura K 543
Ichimura T 1682
Ikeda Y 1892
Ikram MS 1553
Iles RK 1553
Imamura M 429
Imeson JD 1396
Ings SJ 278
Ingvar C 1593
Innocenti F 905
Inoue T 675
International Bone and Cancer
Study Group 1381
Iolascon A 1171
Irrissuto C 480
Ishii H 1717, 1814
Isonishi S 34
Ito I 1415
Ito Y 1211
Izzo A 1171
Jackler RK 998 (L)
Jackson RT 241
Jacobs MV 1421
Jain RK 1513
Jaiswal AK 1305
Jakobisiak M 1485
James WH 2025 (L)
Janeczko M 550
Janicot M 642
Janik P 1041
2026 Author index
British Journal of Cancer (2000) 82(12), 2024-2031 (c) 2000 Cancer Research Campaign
Janot F 642, 1614 (L)
Jansen CT 248 (L)
Jansen RLH 772
Jarrett RF 1117
Jay G 1959
Jayasurya A 1198
Jayne DG 1233
Jayson GC 760
Jemal A 1875
Jenkins V 1783
Jeong S-Y 1403
Jezek J 1808
Jiang W 118
Jimenez E 1662
Jimenez OM 20
Jimeno J 1732
Jockel KH 227
Joensuu H 777
Johansson R 1332
Johnsen R 1358
Johnson TM 657
Johnston A 1519
Johnston PG 560
Johnston SRD 812
Joly F 263
Jonas SK 1004
Jonges TGN 931
Jonker DJ 1789
Jonsson N 1593
Joseph J 20
Joseph P 1305
Jowitt SN 1261
Jung K 1611 (L)
Jung M 1611 (L)
Jung PM 1403
Jung S-M 1953
Kaganoi J 429
Kaghad M 823
Kahlos K 1022
Kaijen P 1123
Kaina B 629
Kainulainen T 1433
Kainz C 1138
Kairemo KJA 777
Kaisary AV 1671
Kakolyris S 844
Kalinski T 1851
Kalipciyan M 1276
Kallincos NC 945
Kambouchner M 412
Kameoka S 404
Kaminski R 1485
Kamradt MC 1709
Kanai T 1717, 1814
Kanazawa M 518
Kane E 1117
Kaneko C 518
Kaneko Y 1801
Kaneyoshi T 833
Kanno H 1446
Kano M 429
Kaprio J 248 (L)
Karabon L 621
Kariyama K 833
Karjalainen JM 2017
Karner J 98
Karner-Hanusch J 1276
Karpinska G 579
Kasajima T 404
Kashiwagi K 1814
Kato H 459, 981
Kato K 891
Kato T 404
Katsikas M 300
Kaur S 1004
Kawabata K 467
Kawaguchi H 1892
Kawaguchi T 418
Kawahara M 418
Kawahara S 104
Kaye SB 392
Ke YQ 1694
Keilholz U 118
Keim V 1013
Keizer HJ 1539
Kelland LR 436
Keller G 315
Kellokoski JK 1983, 2017
Kelsey S 2024
Keng PC 937, 1223
Kerin MJ 1312
Kerr B 1519
Kersey JH 234
Kew TY 1163
Kim JC 1403
Kim KW 385
Kim NK 1403
Kimura T 404
King J 167
King M-C 705
Kinlen LJ 999
Kinnula VL 1022
Kinoshita Y 88
Kirby JA 1900
Kirkwood JM 1755 (L)
Kitamura K 1892
Kitchener HC 760
Kiura K 104
Kjellberg L 1332
Klar E 157
Klijn JGM 151
Klimczak A 621
Klocker H 39
Knekt P 731, 1107
Kobayashi M 838, 953, 1689
Kobayashi Y 833, 838
Koch CJ 937
Kocha W 1789
Koeffler HP 452
Koehler A 931
Koepsell H 629
Kogner P 1561
Kohno N 374
Kohshi K 88
Koizumi T 1682
Kolonel LN 1867
Kondo H 459
Konopa J 1300
Konopinski R 579
Koomagi R 1747
Koot VCM 1017
Korbelik M 1835
Kornek GV 98
Kos J 782
Koshiuka K 452
Koskenvuo M 248 (L)
Kosma V-M 1983
Kosma VM 2017
Kosmas C 300
Kotz R 85
Koukourakis M 844
Koukourakis MI 1427
Kowalczyk P 1041
Kozar K 1485
Kramar A 1965
Krammer P 1851
Krasovec M 782
Kraszewska E 579
Krauss G 98
Kravdal O 737
Kreienbrock L 227
Kreuzer M 227
Kroesen BJ 472
Kronqvist P 1656
Krueger E 1709
Kruit WHJ 772
Kryczek I 621
Ku J-L 1403
Kujala HE 2017
Kumar A 960
Kumar D 1009
Kumar S 1393
Kunisue H 46
Kunkler IH 1247
Kunugita N 88
Kuopio T 1656
Kuppen PJK 1539
Kupryjanczyk J 579
Kurdoglu M 249 (L)
Kurebayashi J 46
Kurek R 1650
Kuriaki Y 891
Kurian KM 1247
Kurosumi M 46
Kurttila E 1022
Kurtz JM 1131
Kusterer K 1650
Kusumoto M 1819
Kusuzaki K 1327
Kutoh E 1123
Kwaan HC 1702
Kwasny W 98
Kwon YG 385
La Vecchia C 204, 1860
Lacava J 560
Lacave R 412
Lachkar S 142
Lacroix J 157
Lage H 488
Lah TT 782
Lahodny J 1138
Lahousen M 1030
Lakhani SR 568
Lal G 1646
Lalisang RI 1914
Lambert J 1051
Lang SH 990
Langbauer G 1249
Lange A 621
Larjava H 1433
Lasek W 1485
Lasser P 871
Late Effects Group of the United
Kingdom Children's Cancer
Study Group 1636
Lau WY 497 (L)
Laurent-Puig P 913
Laux DE 514
Lavail R 1965
Lawrence D 1117
Le Bihan M-L 871
Leake R 1625, 1627
Lebailly P 263
Leblanc-Talent P 171
Lee C 1519
Lee H 852
Lee H-S 852
Lee J 937
Lee OH 385
Lee SM 1158
Lee YM 385
Leek RD 844
Lefrere I 871
Lehnert M 538
Leigh IM 844
Leitz W 220
Lemerle J 1939
Lemoine NR 1058
Lendeckel U 1795
Leonard JH 823
Leone B 560
Leone G 1610 (L)
Lequeux N 1965
Leridant AM 1614 (L)
Lerner K 1433
Lessor T 683
Leung PCK 1415
Leung WT 497 (L)
Leverett D 278
Levesque MA 361
Levi F 204
Levitt NC 1759, 1776
Levy C 818
Lewis IJ 1339
Li Z 429
Liang B 1519
Liao C-T 1953
Libroia A 1772
Lidereau R 142
Liede A 705
Liff J 718
Ligot L 1939
Lim S 538
Lin P 852
Lin S-R 1035
Linch DC 278
Lindh J 601
Lindhorst E 1650
Linet MS 234
Lister TA 2024
Little J 1863
Littlewood TJ 1776
Liu B 1993
Liu G 1646
Livant DL 666
Liyim D 1186
Lluis F 535
Lobo F 1266
Locker G 98
Loening SA 1611 (L)
Logue JP 1177
Loibner H 441
Loidi L 584
Loimas S 900
Lomax ME 1145
Lombardi A 348
Lopes A 1348
Lord EM 937
Author index 2027
British Journal of Cancer (2000) 82(12), 2024-2031
(c) 2000 Cancer Research Campaign
Lorenz I 1851
Lorigan P 1158
Lorigan PC 81, 1547
Lu Y 1993
Luboinski B 1614 (L)
Luisi A 270
Lund Nilsen TI 1358
Luppi M 702
Lustenberger P 1283
Lutz J-M 1111
Lykkesfeldt AE 1844
MacCallum J 1629
McCaw DL 1297
McCormick C 881
MacGregor A 247 (L)
Mach RH 1223
Machaj E 1485
Machiavelli M 560
Machida D 571
McIntyre IA 1469
MacKay EH 186
McKeage MJ 966
McKean-Cowdin R 1867
McKenna SL 1827
McKenzie K 742
MacKenzie MAF 1247
McKeown SR 1469, 1925
McKinna A 568
McMichael AJ 1058
MacMillan A 278
McNally RJQ 1571
McRae CU 241
Mader RM 1276
Maehama T 666
Maehara N 1819
Mafune K-i 1557
Magarey C 167
Magdalena C 584
Magener A 157
Maggi CA 480
Maggioni A 295
Magklara A 361
Magnani P 616
Mahotka C 1851
Mairs RJ 74
Maitland NJ 990
Mak I 812
Makinen K 900
Malamos NA 300
Malfertheiner P 1795
Mali WPThM 1017
Malik KTA 323
Mallone S 227
Malvy D 263
Maly K 39
Mamelle G 1614 (L)
Mancuso P 524
Mandyam R 635
Mangili G 616
Mangioni C 295
Mankin HJ 1327
Mann JR 1568
Mannermaa AJ 2017
Mansi JL 1459
Manzini S 480
Marchetti A 905
Marcuello E 535
Marczell A 98
Marenbach K 1662
Mariani CDA 616
Mariani-Costantini R 348
Marinelli A 1539
Markwart SM 666
Marossy A 568
Maroun JA 1789
Marr P 167
Marras V 553
Marriott JB 1009
Marshman E 924
Martelli G 270
Marth C 1138
Martinelli G 524
Martinez B 1266
Martinez N 1662
Marx D 1662
Masaki T 9
Masback A 1593
Massarelli G 553
Masson D 1283
Massoud C 263
Matano E 1772
Mathieu S 1553
Matsuda T 9, 459
Matsuda Y 1689
Matsunaga K 467
Matsuura N 1211
Matthews J 608
Mattioli S 865
Mattis A 1204
Mattsson A 220
Mazumdar M 550
Meade-Tollin LC 931
Meden H 1662
Mehle C 601
Meijer CJLM 1421
Meijer DKF 472
Meijers-Heijboer H 151
Melbye M 1070
Melia J 1605
Mellemkjaer L 1353
Mendelsohn J 1993
Mendez E 1568
Menger MD 794
Merletti F 227
Mertens AC 234
Messerini L 348
Meyer T 1535
Mezzelani A 1271
Mezzetti M 524
Micca PL 1764
Micek M 705
Middleton MR 1158, 1755 (L)
Middleton SB 827
Miehlke S 1795
Milan T 248 (L)
Milano G 171
Miller WR 136, 1629
Minegishi T 1415
Miner C 1145
Minguell JJ 1290
Minna JD 1191
Mitchell PJ 1983, 2017
Mitsui Y 1974
Mittermayer C 1407
Miwa H 1446
Miyakawa A 543
Miyakawa R 404
Miyake M 374
Miyakoshi J 28
Mizumoto K 1819
Mizutani S 1945
Mohideen N 1709
Moisio KI 1983, 2017
Molema G 472
Monaghan J 1348
Monden M 1211
Moon EJ 385
Mooney R 1740
Mora O 1254
Moran JP 937
Mori A 524
Mori H 467
Mori M 1557
Morita M 1892
Moriya T 518
Morris AG 1510
Morris DL 167
Morris GM 1764
Morrison E 1233
Morrison GH 1764
Moscatello DK 186
Moslinger R 1249
Moss S 1605
Mossner J 1013
Mothersill C 1740
Mrzyk S 1063
Muche JM 1611 (L)
Mueller K-M 763
Mukai M 1814
Mulder GJ 1539
Muller M 1851
Muller-Holzner E 1138
Munakata S 1446
Munday GR 1381
Muradore I 1732
Murakami H 1677
Murata H 1327
Murnane MJ 1317
Murphy PA 1879
Murray MM 1469
Muto T 9
Mutton K 1261
Nabers J 806
Naeyaert JM 823, 1051
Nagano H 1211
Nagashima S 981
Nagesh K 1261
Nagy Z 1441
Naka N 418
Nakahara M 1211
Nakamata T 1677
Nakamori S 1211
Nakamura M 1211
Nakamura T 1677
Nakao K 1211
Nakatsukasa H 833
Nakayama T 1677
Nakazawa A 1717
Nambooze S 1585
Namer M 171
Nanus DM 550
Nardelli F 480
Nardini D 905
Narisawa R 1689
Narod SA 705, 1646
Nash A 568
Naundorf H 1844
Neefs JM 1123
Neglia JP 234
Negri E 204, 1860
Nekulova M 1757 (L)
Neshasteh-Riz A 74
Nessler-Menardi C 39
Neumann C 1844
Newell DR 924, 1519
Newland AC 2024
Newlands ES 608
Newton CJ 1312
Nicholson RI 501, 1163
Nicoletti G 1254
Niggli FK 1239
Nilsson I 1387
Nishida J 891
Nishikawa T 404
Nishimura R 1682
Nishio S 1819
Nishiwaki Y 800
Nogues C 142
Norman A 1959
Norrback K-F 601
Norrish AE 241
Nortier JW 1914
Nosarti C 742
Nose T 1974
Nostro MC 178
Nouso K 833
Nozawa Y 16
Nuutinen P 900
Obiezu C 361
O'Brien MER 93
O'Byrne K 1759, 1776
O'Byrne KJ 1427, 1895 (L)
O'Doherty CA 2024
O'Dwyer ST 7
Oefner P 1249
Oehler MK 749
O'Flaherty E 1900
Ogawa T 1819
Ogawara M 418
Ogg GS 1058
Ohi R 1801
Ohkawa K 34
Ohno S 1557, 1892
Ohno T 16
Oka M 1677
Okayama Lung Cancer Study
Group 104
Okayasu I 571
Okishio K 418
Okui T 838
Okuyama T 800
Olapade-Olaopa EO 186
Ol
/dak T 1485
Olive PL 635
Oliver RTD 2024
Olmeo N 553
Olopade OI 705
Olsen JH 1353
Olsson H 1593
Ongenae N 1123
Onishi T 833
Oomen MH 112
Oppenheim D 251
O'Reilly SM 608
Osanto S 772
Osman S 608
Osorio A 1266
2028 Author index
British Journal of Cancer (2000) 82(12), 2024-2031 (c) 2000 Cancer Research Campaign
Ossu Rocca PC 553
Ostrowski J 1041
Osundeko O 354
Otagiri N 1801
Otsuki T 46
Otsuki Y 1446
Owen J 1158
Owens J 74
Paakko P 1022
Paganelli G 295, 616
Palicio M 535
Palma C 480
Palmer BD 966
Palmer K 124
Palmieri G 553
Palmirotta R 348
Palomba G 553
Panigone S 1945
Panou C 52
Paolucci M 524
Papandreou CN 550
Pappas TN 691
Paradis A-J 1646
Paradiso A 560
Parish D 492
Park J-G 1403
Park YJ 1403
Parkes SE 1568
Parkin DM 1585
Parkins CS 1835
Parliament MB 635
Parma J 315
Parsons MA 330
Pasca A 553
Pastorek J 1808
Patel H 1764
Patterson AV 651
Patterson LH 1469, 1925
Paul J 1932
Payne JT 1297
Pecorelli S 616
Peelen T 151
Peer PGM 381
Peeters PHM 1017
Peinado MA 535
Pelgrims J 291
Pelloni A 1131
Pendry L 1605
Perez ML 1740
Perone P 657
Perry MR 514
Perry SL 1233
Persico I 553
Petersen I 65
Petersen S 65
Peto J 1111
Petroni S 560
Pettit SJ 1900
Peyrottes I 171
Phillips HA 136
Phillips RKS 827
Piccinini EE 56
Piccinini L 1254
Pierotti MA 1271, 1945
Piersma H 1920
Pietsch T 1561
Pignon J-P 871
Pijl MEJ 1539
Pike MC 1867, 1879
Pilkington GJ 52
Pilotti S 1271
Pilz L 591
Pinder SE 1163
Pinkerton CR 1396
Pirastu M 553
Pires S 323
Pirianov G 1459
Pisano A 553
Pisano M 553
Pistorius G 794
Pittermann E 1249
Pivot X 529
Pohl J 629
Poiree B 263
Pol RP 1421
Polak-Charcon S 195
Pollan M 1662
Pollock AM 712
Polyzos A 300
Pope ER 1297
Pope V 718
Potten CS 178
Potter AM 330
Potter JD 234
Pottern LM 718
Poupon M-F 913
Powell JE 1568
Powlesland RM 323
Pozcharskaya V 315
Preston SR 124
Price PM 608
Primrose JN 124
Probst-Hensch NM 1867
Proebstle TM 118
Propper DJ 1759, 1776
Prove A 291
Provencio M 1183
Prudente S 1945
Puente JL 584
Purdie D 1204
Purohit A 492
Pyle L 812
Rabkin C 718
Radford JA 789
Radloff GA 753
Rahman N 538, 568
Rajagopalan B 1776
Ramp U 1851
Rampling R 74
Read LC 945
Rebhandl W 1758 (L)
Reed MJ 492, 1577
Rees RC 330
Reeves JR 74
Regauer S 1030
Reich O 1030
Rembiszewska A 579
Remes K 601
Renehan AG 7
Renneberg H 1650
Rennie IG 330
Renwick A 705
Reyes E 1611 (L)
Reynolds K 760
Reynolds PA 323
Reza MS 74
Rezvani M 1764
Riccardi A 1254
Ricci S 412
Ridwelski K 1795
Riggi M 524
Rinaldi E 1254
Rishi AK 683
Rissanen H 1107
Ritchie LD 1863
Rivera F 20
Rizzoli R 858
Roberge C 263
Roberts JV 742
Roberts KG 1671
Robertson C 1887
Robertson JD 1261
Robertson LM 74
Robinson D 1111
Robinson SP 2009
Robison LL 234
Robledo M 1266
Roddiger S 1650
Rodriguez FJ 514
Roesch F 227
Rofstad EK 1528
Rogers P 436
Romero A 560
Ronsin M 1939
Ronzoni S 1732
Rooprai HK 52
Roos G 601
Rose C 339
Rosenquist R 601
Ross JA 753
Ross RK 1364
Rostagno P 171
Rosty C 913
Rougier P 871
Rubenstein A 998 (L)
Rucklidge GJ 52
Ruers TJM 381
Rumi C 1610 (L)
Runsink AP 1421
Ruparelia K 1925
Rusin M 579
Rustin GJS 1535
Rutella S 1610 (L)
Rutqvist LE 220
Rybczynska J 1485
Ryder WDJ 760
Saarikoski S 1983
Sabin C 702
Sabourin J-C 871
Saegusa M 571
Saeki H 1892
Sage RD 446
Said JW 452
Sakae K 104
Sakon M 1211
Sakurai T 429
Salgado R 1896 (L)
Salo T 1433
Sampson FC 81
San Roman JM 1266
Sandhu DPS 186
Sandstedt B 1561
Sanoudou D 1218
Santoro M 315
Santos C 635
Sapunar F 812
Sarasin A 308
Sarobba MG 553
Sasaki S 9
Sasano H 518
Sato F 429
Sato N 1819
Saunders MP 651
Sauter ER 361
Savage P 1058
Saviozzi S 1945
Scarffe JH 1261
Schadendorf D 488, 1453
Schafer BW 1239
Schally AV 1724
Scheithauer W 98
Schiefke I 1013
Schittek B 1149
Schlienger P 818
Schlumberger M 308
Schluns K 65
Schmidt EE 543
Schmidt M 1993
Schmidt T 657
Schneider J 1662
Schoenberg JB 718
Scholefield JH 960
Scholl FA 1239
Schouten HC 1914
Schratter-Sehn A 98
Schrijvers D 291
Schroder FH 283
Schuder G 794
Schumann T 1013
Schumm-Draeger PM 1650
Schuster K 399
Schwartz AG 718
Schwartz B 195
Schwartz P 167
Schweiger A 782
Sciot R 823
Screnci D 966
Seal S 568
Sedivy R 1276
See L-C 1953
Segawa Y 104
Seifert M 1249
Seker H 629
Selvan RS 691
Senaratne SG 1459
Sengupta PS 760
Severi G 1887
Severson RK 234
Seymour CB 1740
Seymour K 1900
Seynhaeve ALB 973
Shaddick G 1103
Shalet SM 789
Shamash J 2024
Shanks JH 760
Sharp L 1863
Sharp SY 436
Shen R 550
Sheridan MT 1177
Shibuya H 1835
Shimada Y 429
Shimizu K 16
Shimizu N 46, 1801
Shinozaki H 1415
Shiu M-N 1871
Shiwaku HO 1801
Shu XO 234
Author index 2029
British Journal of Cancer (2000) 82(12), 2024-2031
(c) 2000 Cancer Research Campaign
Shuja S 1317
Sica S 1610 (L)
Sideri M 295
Silva JM 1183
Silvestrini R 270
Simonato L 227
Simone G 560
Simony-Lafontaine J 1965
Sisley K 330
Skiner R 1636
Skladanowski A 1300
Slade RJ 760
Sleijfer DTh 806
Smedsrod B 953
Smeets JBE 806
Smets EMA 789
Smibert E 255
Smith DR 1764
Smith IE 812, 1907
Smith M 1932
Smith PG 924
Smith RE 931
Snary D 1671
Sng J-H 538
Snieder H 247 (L)
Snijders PJF 1421
Sobczak-Thepot J 642
Sobel A 142
Sodha N 1145
Soini Y 1022
Songini C 616
Sonoo H 46
Sorensen FB 339
Soufir N 818
Soukop M 1932
Soulie P 913
Soutar DS 392
Southern SA 424
Sozzi G 1271
Spanedda R 1254
Spataro V 1131
Spector T 247 (L)
Speirs V 1312
Speleman F 823
Spence HJ 1615
Spierings DCJ 1474
Spinelli R 767
Spitz L 250 (BR)
Sprangers MAG 1131
Springer E 1063
Srinivasan R 1163
Staib A 782
Stamato TD 1740
Stanczyk F 1879
Stanczyk FZ 1867
Standiford SB 514
Stanisch M 1650
Stapleton G 1615
Steel CM 705, 1247
Steger GG 1276
Stelmachow J 579
Stevens MCG 1636
Steward LMD 1671
Steward WP 1427
Stewart AFR 446
Stewart THM 446
Stiller CA 1339
Stockton D 208
Stokl
/osa T 1485
Stolte M 1795
Stone JG 568
Stoppa-Lyonnet D 818
Stower M 990
Stratford IJ 651, 1177, 1925
Stratford MRL 1835
Stratton M 151
Stratton MR 568
Strickler H 718
Strieth S 591
Stubbs M 2009
Stummvoll W 1138
Suarez HG 308
Sugie S 467
Sugimachi K 1557, 1892
Sugimoto T 1682
Suginoshita T 1327
Sujansky E 998 (L)
Suminami Y 981
Sutherland DJA 361
Suzuki T 518
Suzuki Y 1689
Svoboda K 1702
Swanson GM 718
Sweetenham JW 4
Syrjanen K 1983
Szamel I 492
Szende B 1724
Szepeshazi K 1724
Szymanska G 579
Tabata M 104
Tada Y 452
Tagliabue E 1945
Tagliaferri P 1772
Takano S 1974
Takeda T 1211
Takeshita H 1327
Taki T 374
Talamini R 1860
Talbot DC 1759, 1776
Talbot M 1614 (L)
Tamborini E 1271
Tan NG 1198
Tan W 538
Tanaka K 46, 800
Tanaka M 1819
Tanaka S 1557
Tanaka T 34
Tanda F 553
Tang CK 46
Tanimoto K 838
Tate D 1297
Tavani A 1860
Taya Y 1945
Taylor GM 1117, 1571
Taylor M 844
Tealdo F 524
Teissier E 171
ten Hagen TLM 973, 1000
Tenhunen M 777
Teppo L 248 (L), 1107
Terao Y 891
Terry G 1348
Terry TR 186
Tetlow L 354
Tewarie L 381
Thatcher N 1158
Thomas BS 1577
Thomas GA 315
Thompson CH 1776
Tidefelt U 1387
Tilanus HW 1510
Tilanus MGJ 1510
Timmerman MA 112
Timorek A 579
Tindall DJ 361
Tinelli C 1254
Tingby O 1218
Toguchida J 1677
Toh CH 1694
Tokuchi Y 838
Tokus M 399
Tollenaar RAEM 1539
Tomasic G 270
Tomei F 204
Tomono Y 1974
Tompson RV 1297
Tonelli F 348
Tonini GP 1171
Tonnachera M 315
Tookman A 1485 (BR)
Torchilin VP 1513
Torelli G 702
Tortola S 535
Tosaki T 88
Toshikuni N 833
Tran M 263
Treweeke AT 1694
Trieb K 85
Tronko ND 315
Trubetskoy VS 1513
Trzeciak L 1041
Tsai J-H 1035
Tsai K-J 852
Tsavaris NB 300
Tsuboi K 1974
Tsuboyama T 1677
Tsuchiya E 838
Tsuda H 675
Tsuji T 833
Tsujimoto M 1211, 1446
Tuomilehto J 731
Turk V 782
Turley H 1427
Turner HE 1441
Tursz T 642
Uchitomi Y 800
Uchiyama I 675
Ueno H 891
Ueoka H 104
Ueoka Y 891
Uges DRA 767
Ugolini G 56
UKCCR Elderly Cancer Patients
in Clinical Trials Working
Group 1
Umesaki N 675
Umeshita K 1211
Umetani N 9
United Kingdom Childhood
Cancer Study Investigators
1073
United Kingdom Children
Cancer Study Group
(UKCCSG) 1396
Urashima T 1819
Urban T 412
Urdl W 1030
Usadel KH 1650
Uscinska B 213
Usmani BA 550
Vabre L 1614 (L)
Vach W 339
Vahrmeijer AL 1539
Valanzano R 348
Valentini D 1254
Valero V 529
Vallejo C 560
Valota O 767
Van Belle S 823
van Bockel JH 1539
Van Camp B 953
van de Velde CJH 1539
Van den Brande J 291
van der Kwast TH 112
van der Pool S 538
van der Sluis RF 381
van der Veen AH 973
van Dierendonck JH 1539
van Diest PJ 368
Van Gele M 823
van Herpen CML 772
Van Marck E 1051
Van Noorden CJF 931
van Ommen G-JB 151
van Oosterom AT 767
van Putten JWG 806
Van Riet I 953
Van Roy N 823
van Schaik RHN 112
van Steenbrugge GJ 112
van Tiel ST 973, 1000
van Vliet M 151
van Vroonhoven ThJMV 1017
van Weerden WM 112
Vancoillie G 1051
Vande Broek I 953
Vanderkerken K 953
Varani J 657
Varga JL 1724
Varmeh-Ziaie S 543
Vasen HFA 151
Vassal G 642
Vatten LJ 1358
Vaughan ATM 1709
Vaughan MM 812
Vecchio G 315
Vegazo IS 1183
Velek J 1808
Veneroni S 270
Verkasalo PK 248 (L)
Verkooijen HM 1017
Vermeulen PB 1896 (L)
Vermorken JB 291
Verweij J 767
Vesprini D 1646
Vickers N 712
Vignati S 1732
Voest EE 1914
Voit C 118
Vollmar B 794
Volm M 1662, 1747
von Knebel Doeberitz M 157
von Schweinitz D 1561
Voorhorst FJ 1421
Voscoboinik L 315
Vujanovic NL 981
2030 Author index
British Journal of Cancer (2000) 82(12), 2024-2031 (c) 2000 Cancer Research Campaign
Author index 2031
British Journal of Cancer (2000) 82(12), 2024-2031
(c) 2000 Cancer Research Campaign
Wabinga HR 1585
Wabwire-Mangen F 1585
Wadell G 1332
Wager E 93
Wagner TMU 1249
Wake N 459, 891
Walboomers JMM 1421
Wald NJ 731
Wallen CA 1223
Wals J 1914
Walter S 1709
Walton DS 1312
Wan P 1879
Wang DY 492, 1577
Wang H-J 852
Wang J 1702
Wang J-Y 1035
Wang L-M 1223
Wang S-L 852
Wang T-CV 1953
Wang XW 683
Ward A 568
Ward FE 691
Warner E 705
Wa
sik M 1485
Wass JAH 1441
Watanabe G 429
Watanabe K 459
Watanabe M 1717, 1814
Watanabe T 9
Watson GJ 1671
Watson SA 960
Watt HC 731
Watts MJ 278
Weber B 705
Weber L 118
Weber T 157
Wegener K 763
Weiskop S 255
Weiss RA 702
Weissgerber G 1063
Weitz J 157
Welker P 1453
Wen W 234
Wen-yi Y 16
Wenzel M 1851
Werle B 782
Werner M 1204
Wersall P 246 (L)
Wesch H 763
West CML 1177
West DC 1694
West MK 1297
Westerdahl J 1593
Weytjens R 1896 (L)
Whateley TL 74
Wheeler KT 1223
Whelan P 283
Whitby D 702
Whiteside TL 981
Whitley E 227
Wicker R 308
Wideman CL 74
Wierikx CDJ 112
Wieler M 629
Wijnhoven BPL 1510
Wiklund TA 777
Wilczynski G 1485
Willemse PHB 1920
Willemsen EL 492
Williams ED 315
Williams GH 315
Williamson E 452
Wils JA 1914
Wilschut J 1474
Wilson JW 178
Wilson P 1932
Windbichler GH 1138
Winter R 1030
Witzigmann H 1013
Wobbes T 381
Wocial T 1041
Wohlfahrt J 1070
Wolf B 1276
Wolokoff B 635
Wong AJ 186
Wong J 538
Woods Ignatoski KM 666
Woszczynski M 1041
Wright DH 1117
Wu AH 1879
Wu C-H 1035
Wyllie AH 1247
Wyshak G 726
Xia X-M 683
Xian CJ 945
Yagi K 28
Yajima T 1717
Yamada T 1945
Yamada Y 467
Yamamoto H 1677
Yamamoto K 404, 675, 833
Yamamoto S 46, 418
Yamazaki M 1717
Yan PS 514
Yandell DW 579
Yang C-C 1480
Yang Q 763
Yang S-D 1480
Yap WM 1198
Yokosaki Y 1211
Yokota A 88
Yoo J-Y 683
Yoon K-A 1403
Yoshiga K 838
Yoshikawa Y 459
Yoshimi N 467
Young CYF 361
Young H 608
Young IE 1247
Yu J 1795
Yu MC 1364
Yuan F 1513
Yuan J-M 1364
Zagozdzon R 1485
Zambon M 1261
Zambon P 227
Zarandi M 1724
Zavada J 1808
Zavadova Z 1808
Zeigler ME 657
Zekri J 858
Zhang C 1879
Zhou Y 459
Zielinski C 1249
Zielinski D 1421
Zimmerman PV 1191
Zucchetti M 524

				

